# To which non-physician health professionals do French general practitioners refer their patients to and what factors are associated with these referrals? Secondary analysis of the French national cross-sectional ECOGEN study

**DOI:** 10.1186/s12913-021-07285-4

**Published:** 2022-01-05

**Authors:** Matthieu Peurois, Matthieu Chopin, Gaëlle Texier-Legendre, Cécile Angoulvant, William Bellanger, Cyril Bègue, Aline Ramond-Roquin

**Affiliations:** 1 Département de médecine générale, Univ Angers, F-49000 Angers, France; 2 Univ Angers, Univ Rennes, EHESP, Inserm, IRSET-ESTER, SFR ICAT, F-49000 Angers, France; 3 Département de médecine de famille et de médecine d’urgence, Université de Sherbrooke, Québec, Canada

**Keywords:** Primary care, Health workforce, General practice, Referral, Health care organisation, Management

## Abstract

**Background:**

Multiprofessional practice is a key component in primary care. Examining general practitioner (GP) referral frequency to non-physician health professionals (NPHP) can provide information about how primary care is organised and works which is useful for policymakers. Our study aimed to describe French GP referral frequency to various NPHPs in France and identify associated factors.

**Methods:**

This is an ancillary study to the observational, cross-sectional (ECOGEN) study conducted in 2011/2012 in France among 128 GPs. Data about consultations using the standardised International Classification of Primary Care (ICPC-2), and patient and GP characteristics were collected from 20,613 GP consultations. Referrals were identified through inductive and deductive approaches using ICPC-2 codes, keywords, and deep, open manual searches. Referral frequency was described overall and per NPHP. Patient, GP, and consultation-related factors associated with referral rates were described for the three most frequently identified NPHPs. To minimise potential sources of bias, this observational study followed the STROBE guidelines.

**Results:**

French GPs referred 6.8% of patients to NPHPs, with physiotherapists, podiatrists, and nurses accounting for 85.2% of referrals. Older patients, retired patients, multiple health problems managed, and longer consultation durations were found to be associated with higher referral rates (*p* < 0.001). Specific trends were observed for nurse, physiotherapist, and podiatrist referrals. Women (*p* < 0.001) and regular patients (*p* = 0.002) were more likely to receive physiotherapy referrals while people with no professional activity were less likely (*p* < 0.001). Female GPs and those working in urban practices were more likely to issue a physiotherapy referral (*p* < 0.001), while GPs working in rural practices (*p* < 0.001) and those with higher annual consultation numbers (*p* = 0.002) were more likely to refer to a nurse. Working in multiprofessional centres appeared to have little impact on referral rates, being only slightly associated with podiatrist referrals (*p* = 0.003).

**Conclusions:**

Referral frequency is more associated with patient characteristics and clinical situations than GP-related factors suggesting patients needing referral most are most often referred. Furthermore, the three NPHPs that GPs refer to the most are those for which a referral is required for reimbursement in France, suggesting that health system legislation and NPHP reimbursement are strong determinants for referrals.

## Background

The increasing prevalence of chronic diseases, multimorbidity [1, 2], and widening health needs [3, 4] is creating challenges in primary care, causing it to continually expand and change. In the context of this changing role, well-organised primary care helps reduce costs, improves user satisfaction, population health outcomes and equity, and strengthens health system performance [5]. Considering this, numerous countries have initiated health system reforms built around restructuring primary care provision, integrating patient care pathways, and enhancing interprofessional collaborations [6–9].

General practitioners (GP) play a key role in most health care systems since they are often responsible for coordinating patient care pathways, including referrals to non-physician health professionals (NPHPs) [10] and both the primary and secondary care sector [11]. In some countries they act as gatekeepers with GP referral validating health insurance reimbursement for consultations with other health professionals, including some NPHPs.

GP referral to NPHPs is a key indicator for multiprofessional practice [12]. Examining GP referral frequency to different NPHPs, and factors associated with referrals, can provide information about how primary care is organised and works.

Despite growing interest in developing multiprofessional practice in primary care, literature about NPHP referrals in general practice is still very scarce, apart from studies on nurse referrals or referrals for specific chronic diseases [13–17]. This knowledge would be useful for policymakers and all those involved in planning for future healthcare workforce requirements and developing multiprofessional practice in primary care teams [12].

NPHP referrals in general practice probably differ depending on the health care system. Therefore, any data on this topic should be carefully interpreted according to the national context, before being compared with data from other countries when available. In France, as in many other health care systems, GPs, nurses, and pharmacists form the core of primary care, along with various other health professionals, such as dentists, physiotherapists, midwifes, or podiatrists [18, 19]. A 2009 paper described the French primary care system as a “professional non-hierarchical model”, like Germany or Canada, characterised by low level territorial organisation, coexistence of different practice types (solo practices, mono- and multiprofessional groups, including GPs, specialists, and NPHPs) and mainly private practices. This description was compared with “professional hierarchical models”, where GPs have a strong, long-standing, and more formal gatekeeping role (such as in the UK, Netherlands, Australia, or New-Zealand) or “normative hierarchical models”, where primary care is legally defined and based on multiprofessional territorial organisations (such as in Catalonia in Spain, Finland, or Sweden) [20]. Since then, French health policies and professional leaders have supported implementing multiprofessional practice and organisations in primary care, resulting in approximately 15% of GPs practicing within interprofessional health care teams, and a majority being engaged in various forms of multiprofessional practice. Furthermore, in France, GP referrals validate health insurance reimbursement for consultations with some NPHPs such as physiotherapists, nurses, speech therapists or podiatrists, while no GP referral is required to see pharmacists, dentists or midwifes. Except for specific situations, care from dieticians, psychologists or osteopaths is not reimbursed and patients must cover the full cost.

Building on original, nationwide, practice-based observational data, this study aims to describe French GP referral frequency to various NPHPs and identify factors associated with referral rates.

## Methods

### Study design

This study is a secondary analysis of ECOGEN (Elements of Consultation in General practice), a multicentric, observational, cross-sectional, nationwide study, aimed at describing general practice consultations in France. The ECOGEN study design has been previously described [17]. To minimise potential sources of bias, this observational study followed the STrengthening the Reporting of OBservational studies in Epidemiology (STROBE) guidelines.

All procedures performed in studies involving human participants were in accordance with the national research committee and with the 1964 Helsinki declaration and its later amendments or comparable ethical standards.

### Data collection

The ECOGEN study was conducted between December 2011 and April 2012, with an initially expected sample size of 16,000 consultations. Fifty-four trainee GPs from 27 French medical schools were trained to use the second version of International Classification of Primary Care (ICPC-2) [21, 22] which is the standardised classification chosen by the World Health Organisation for primary care [23]. ICPC-2 classifies patient data and clinical activity in terms of reason(s) for consultation (why the patient consulted the GP), consultation result(s) (the diagnosis/problems/health conditions managed during the consultation) and healthcare procedure(s) (any intervention, including referral, performed, or prescribed during the consultation). The trainees collected data from 128 GP internship supervisors. They observed their GP supervisor 1 day/week and systematically collected data for all consultations conducted on that day.

**Fig. 1:**
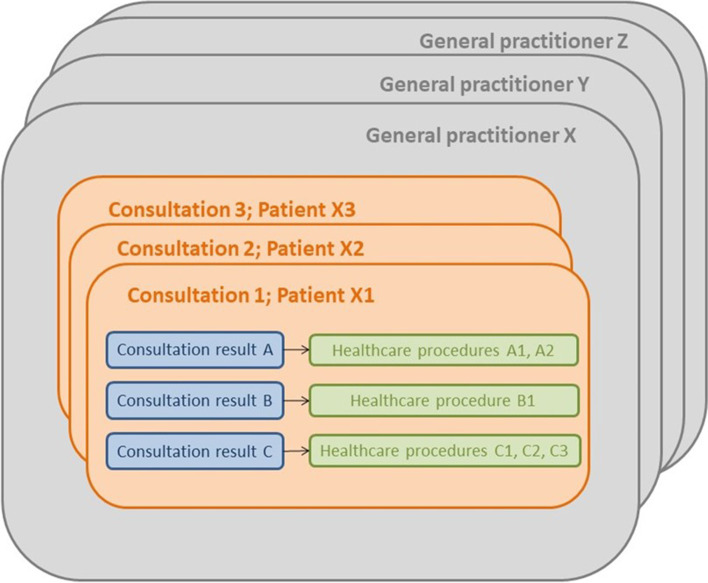
Hierarchical structure of consultation data in ECOGEN study

Specifically, patient data included age, sex, new or known patient, and socio-occupational information. Socio-occupational categories were manual workers (such as builders and joiners), employees (such as office workers), other professional activities (including farmers, craftspeople, retailers, and senior managers), no professional activity (indicating people with no active employment including unemployed people, students, children, stay-at-home parents etc. excluding retired people) and retired. GP data included age, sex, practice location (rural, semi-rural or urban based on the GP’s self-reported subjective response), practice type (solo, mono-professional group, or multi-professional group) and annual practice volume (annual number of consultations). Consultation data included consultation duration, as well as reasons for consultation, consultation results and healthcare procedures using ICPC-2 codes supplemented by verbatim and a hierarchical structure (Fig. 1).

#### Definition of non-physician health professionals (NPHP)

In this study “non-physician health professionals” refer to any registered health professionals except for physicians.

### Data extraction

Inductive and deductive approaches were used to identify GP consultations resulting in patient referral to NPHPs using codes, keywords, and a deep, open manual search. A list of NPHPs and related keywords were initially compiled (Table 1). ICPC-2 codes relevant to our study were then identified. Codes − 66 [referral to another health professional (excluding physicians)], and − 68 [other referrals (not specified elsewhere)] were used in the automatic search strategy, while the code − 67 [referral to physician/specialist/clinic/Hospital] was not. We also considered the code − 57 [physiotherapy / rehabilitation] in relation with manual therapists. Finally, an Excel search with advanced filters was performed using the following 3-step process.

- Step 1: Identify healthcare procedures coded − 66 OR − 68 in ICPC-2 (codes relating to referrals, except to a physician) **with**, then **without**, verbatim including at least one NPHP keyword. The observation (healthcare procedure and all related patient, GP, and consultation data) was automatically selected if verbatim included one or more NPHP keywords from our list. If verbatim did not include any NPHP keywords from our list, observations where referral to an NPHP was clearly stated in the verbatim were manually selected.
- Step 2: Identify healthcare procedures with any code other than − 66 or − 68 and verbatims including at least one NPHP keyword from our list. Only observations whose verbatim clearly related to a patient referral were manually selected.
- Step 3: Identify procedures coded − 57 (code relating to manual therapy) and verbatims not including any of the NPHP keywords from our list. Only observations with verbatim clearly relating to referrals to physiotherapists or osteopaths (the two NPHPS using manual therapy in France) were manually selected.

**Table 1:** First list of NPHPs considered and related keywords

Profession (English)	Profession (French)	Keywords (French)
Nurses	Infirmiers/infirmières	« *infirmi* » or « * ide* »
Physiotherapists	Kinésithérapeutes/rééducation	« *kin* » or « *rééduc* »
Psychologists	Psychologues	« *psychol* » and not « *soutien* »
Midwives	Sage-femmes	« *sage* » or « *sf* »
Dentists	Dentistes	« *dent* »
Dieticians/nutritionists	Diététicienne/nutritionniste	« *diét* » or «*nutri* » or « *diététic* »
Osteopaths	Ostéopathe/thérapie manuelle	« *ostéo* » or « *manuel* » or *manip* »
Social workers	Assistantes sociales	« *assist* » or « *social* » or « *as* »
Pharmacists	Pharmaciens	« *pharma* »
Podiatrists	Podologues/semelles	« *podo* » or « *pédic* » or « *semel* »
Speech therapists	Orthophonistes	« *orthoph* »
Occupational therapists	Ergothérapeutes	« *ergot* »
Psychomotor therapists	Psychomotricien-nes	« *psychomot* »

The data extraction process was mainly conducted by the last author (ARR). In the case of automatic selection of observations (codes − 66 or − 68 AND presence of NPHP keywords in the verbatim), only ARR checked the selection appropriateness. In all the other cases (manual selection, when codes or keywords were absent) a double check (ARR with CB or CA) was undertaken.

### Data analysis

Categorical variables were described as number (%) and continuous variables were described as mean (SD). The referral frequency was described, both overall and per NPHP. Patient, GP, and consultation-related factors associated with referral rates were described for the three most frequently identified NPHPs, using univariate statistical analysis: chi-2 tests in the case of categorical variables, and Student-t tests in the case of continuous variables. Due to the study sample size and the multiple comparisons, only highly statistically significant associations (*p*-values < 0.001) have been highlighted. Analyses were performed using BiostaTGV.

## Results

The ECOGEN database contains 20,613 consultations, 45,582 consultation results and 98,847 healthcare procedures.

### Patient, GP, and consultation characteristics

The average patient age was 46.6 years, 58.2% were women, 32.8% were retired and 94.5% were regular patients. The average GP age was 53 years, 34.3% were women, 54.3% practiced in urban areas, and 79.9% were in a mono-professional practice (either solo or group). The mean consultation duration was 16.7 min, the average number of consultation results (health conditions managed) per consultation was 2.21 and the average number of healthcare procedures per consultation was 4.8 (Table 2).

**Table 2:** Patient, GP, and consultation characteristics in the ECOGEN dataset

	Total (*n* = 20,613)
**Patient Characteristics**	
Age: mean (SD)	46.6 (25.7)
Female: number (%)	11,995 (58.2)
Socio-occupational category: number (%)	
Employee	3972 (19.3)
Worker	815 (4.0)
Other professional activity^a^	2910 (14.2)
No professional activity^b^	6150 (29.8)
Retired	6766 (32.8)
Regular patients: number (%)	19,473 (94.5)
**General practitioner characteristics**	
Age: mean (SD)	53.0 (7.5)
Female: number (%)	7063 (34.3)
Location: number (%)	
Rural	4163 (20.2)
Semi-rural	5266 (25.5)
Urban	11,184 (54.3)
Practice type: number (%)	
Solo	4330 (21.0)
Mono-professional medical group	12,149 (58.9)
Multiprofessional centres	4134 (20.1)
Annual number of consultations: mean (SD)	5162 (1749)
**Consultation Characteristics**	
Mean duration: minutes (SD)	16.7 (8.3)
Number of consultation results: mean (SD)	2.21 (1.44)
Number of consultation results during the consultation	
1 consultation result: number (%)	8567 (41.6)
2 consultation results: number (%)	5644 (27.4)
3 consultation results: number (%)	3250 (15.8)
4 consultation results: number (%)	1614 (7.8)
5+ consultation results: number (%)	1538 (7.5)
Healthcare procedures delivered per consultation: mean (SD)	4.8 (3.09)

^a^Includes professions such as farmers, retailers, craftspeople and senior managers

^b^Includes children, unemployed people, stay at home parents, students etc. but does not include retired people

GPs referred 1396 patients (6.8%) to an NPHP (Table 3). The total number of referrals was 1455 reflecting the fact that some patients were referred to two or more NPHPs during the same consultation. Patients were mostly referred to physiotherapists (4.5%), podiatrists (0.8%) and nurses (0.7%), accounting for 85.2% of referrals.

**Table 3:** Number of patients general practitioners referred to NPHPs

Non-physician health professional	Number of referrals (***n*** = 1455)	% of total consultations
Physiotherapists	927 (63.7%)	4.5
Podiatrists	172 (11.7%)	0.8
Nurses	142 (9.8%)	0.7
Psychologists	49 (3.4%)	0.2
Osteopaths	36 (2.5%)	0.2
Speech therapists	29 (2%)	0.1
Dentists	28 (1.9%)	0.1
Nutritionists	27 (1.9%)	0.1
Others^a^	45 (3.1%)	0.2

^a^Others include social workers, orthoptists, midwives, etc.

### Referrals to physiotherapists, podiatrists, and nurses

Table 4 highlights factors associated with referrals to physiotherapists, podiatrists, and nurses. In general, those who received a referral were older than those who did not. This was particularly true for nurse referrals, where the mean patient age was 70.3 (SD 20.7) years versus 46.4 (SD 25.7) years for those who did not receive a referral. Referrals to physiotherapists, podiatrists, and nurses were strongly associated with longer consultation durations. Consultations with physiotherapy referrals were an average of 2.4 min longer, podiatrists an average of 2.6 min longer and nurses an average of 3.8 min longer.

**Table 4:** Patient, GP, and consultation-related factors associated with GP referral to physiotherapists, podiatrists, or nurses

	Referrals to physiotherapists			Referrals to podiatrists			Referrals to nurses		
	Consultations with referral to a physiotherapist (*n* = 927)	Consultations without referral to a physiotherapist (*n* = 19,686)	*p*-value	Consultations with referral to a podiatrist (*n* = 172)	Consultations without referral to a podiatrist (*n* = 20,441)	*p*-value	Consultations with referral to a nurse (*n* = 142)	Consultations without referral to a nurse (*n* = 20,471)	*p*-value
**Patient characteristics**									
Age: mean (SD)	51.7 (23.2)	46.4 (25.8)	< 0.001*	52.9 (21.8)	46.5 (25.7)	< 0.001*	70.3 (20.7)	46.4 (25.7)	< 0.001*
Female: number (%)	603 (65.0)	11,392 (57.9)	< 0.001*	101 (58.7)	11,894 (58.2)	0.89	90 (63.4)	11,905 (58.2)	0.21
Socio-occupational category : number (%)			< 0.001*			0.03			< 0.001*
Employee	218 (23.5)	3754 (19.1)		32 (18.6)	3940 (19.3)		10 (7.0)	3962 (19.4)	
Manual worker	47 (5.1)	768 (3.9)		4 (2.3)	811 (4.0)		5 (3.5)	810 (4.0)	
Other professional activity^a^	137 (14.8)	2773 (14.1)		29 (16.9)	2881 (14.1)		6 (4.2)	2904 (14.3)	
No professional activity^b^	191 (20.6)	5959 (30.3)		37 (21.5)	6113 (29.9)		21 (14.8)	6129 (29.9)	
Retired	334 (36.0)	6432 (32.7)		70 (40.7)	6696 (32.8)		100 (70.4)	6666 (32.6)	
Regular patients: number (%)	897 (96.8)	18,576 (94.4)	0.002	165 (95.9)	19,308 (94.5)	0.40	136 (95.8)	19,337 (94.5)	0.50
**GP characteristics**									
Age: mean (SD)	52.5 (7.7)	53.0 (7.5)	0.04	51.7 (7.5)	53.0 (7.5)	0.025	53.3 (7.5)	53.0 (7.5)	0.60
Female: number (%)	394 (42.5)	6669 (33.9)	< 0.001*	68 (39.5)	6995 (34.2)	0.14	41 (28.9)	7022 (34.3)	0.17
Location: number (%)			< 0.001*			0.10			< 0.001*
Rural	159 (17.2)	4004 (20.3)		33 (19.2)	4130 (20.2)		54 (38.0)	4109 (20.1)	
Semi-rural	172 (18.6)	5094 (25.9)		33 (19.2)	5233 (25.6)		27 (19.0)	5239 (25.6)	
Urban	596 (64.3)	10,588 (53.8)		106 (61.6)	11,078 (54.2)		61 (43.0)	11,123 (54.3)	
Practice type: number (%)			0.46			0.003			0.01
Solo	214 (23.1)	4116 (20.9)		28 (16.3)	4302 (21.0)		24 (16.9)	4306 (21.0)	
Group	530 (57.2)	11,619 (59.0)		90 (52.3)	12,059 (59.0)		98 (69.0)	12,051 (58.9)	
Multi-professional centre	183 (19.7)	3951 (20.1)		54 (31.4)	4083 (20.0)		20 (14.1)	4114 (20.1)	
Annual number of consultations: mean (SD)	5071 (1693)	5166 (1752)	0.11	5004 (1642)	5163 (1750)	0.24	5619 (1825)	5158 (1748)	0.002
**Consultation characteristics**									
Mean duration: minutes (SD)	19.0 (8.3)	16.6 (8.3)	< 0.001*	19.3 (8.6)	16.7 (8.3)	< 0.001*	20.5 (9.6)	16.7 (8.3)	< 0.001*
Number of consultation results: mean (SD)	2.74 (1.73)	2.16 (1.42)	< 0.001*	2.92 (1.53)	2.18 (1.44)	< 0.001*	2.76 (1.8)	2.18 (1.44)	< 0.001*
Number of consultation results: number (%)			< 0.001*			< 0.001*			< 0.001*
1 consultation result: number (%)	252 (27.2)	8315 (42.2)		24 (14.0)	8543 (41.8)		34 (23.9)	8533 (41.7)	
2 consultation results: number (%)	258 (27.8)	5386 (27.4)		58 (33.7)	5586 (27.3)		41 (28.9)	5603 (27.4)	
3 consultation results: number (%)	176 (19.0)	3074 (15.6)		42 (24.4)	3208 (15.7)		31 (21.8)	3219 (15.7)	
4 consultation results: number (%)	102 (11.0)	1512 (7.7)		23 (13.4)	1591 (7.8)		22 (15.5)	1592 (7.8)	
5+ consultation results: number (%)	139 (15.0)	1399 (7.1)		25 (14.5)	1513 (7.4)		14 (9.9)	1524 (7.4)	

*Indicates statistically significant results

^a^Includes professions such as farmers, retailers, craftspeople, and senior managers

^b^Includes children, unemployed people, stay at home parents, students etc. but does not include retired people

**Fig. 2:**
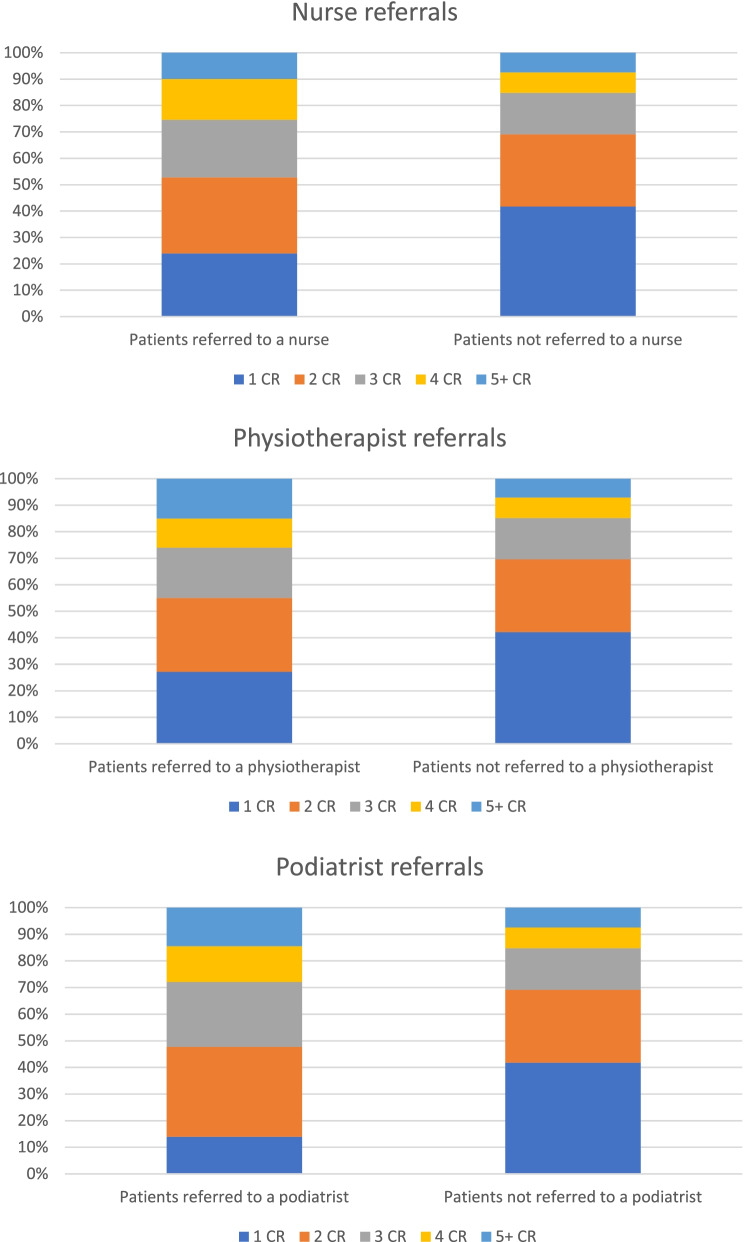
Significant positive association between number of consultation results (CR) and nurse, physiotherapist, and podiatrist referral rates

In addition, 47.2% of patients referred to a nurse had three or more consultation results compared with only 30.9% of patients not referred to a nurse. Similar significant differences were observed with referrals to physiotherapists (45.0% of referred patients have three or more consultation results versus 30.4% in those not referred) and podiatrists (52.3% versus 30.9%) (See Fig. 2).

No significant (*p* < 0.001) associations were found between referrals to any of these three NPHPs and GP age.

### Physiotherapist referrals

In addition to the previously described associations, women were more frequently referred to a physiotherapist, accounting for 65% of consultations resulting in a physiotherapy referral compared with 57.9% with no referral. Regular patients appear to be more often referred than non-regular patients, but this was only a non-significant trend (*p* = 0.002). When comparing people who received a physiotherapy referral and those who did not, people with no professional activity were less frequently referred to a physiotherapist than those in other socio-occupational categories (patients with no professional activity accounted for 20.6% of patients referred to a physiotherapist compared with 30.3% of those not referred). This association remained significant even after restriction to non-retired people. Female GPs were more likely to issue a physiotherapy referral (female GPs accounted for 42.5% of consultations with a physiotherapy referral, compared with 33.9% in consultations with no referral). Furthermore, urban practices account for 64.3% of consultations with a physiotherapist referral compared with 53.8% with no physiotherapist referral.

### Nurse referrals

When comparing patients who received a nurse referral and those who did not, 70.4% of patients referred to a nurse were retired compared with just 32.6% of patients who were not referred. Rural practices appear to be more likely to refer to a nurse (accounting for 38% of consultations with a nurse referral compared with 20.1% with no nurse referral). Furthermore, even though non-significant, GPs with higher annual consultation numbers seemed to be associated with more nurse referrals (an average of 5619 consultations per year for GPs referring a patient to a nurse versus 5158 for those who did not, *p* = 0.002).

### Podiatrist referrals

In addition to previously described associations that are common to all three NPHPs, only a non-significant (*p* > 0.001) trend was observed for podiatrists. GPs in multiprofessional centres appear more likely to refer to a podiatrist (31.4% of GPs referring a patient to a podiatrist worked in a multiprofessional practice versus 20% of those who did not refer (*p* = 0.003)).

## Discussion

Our findings show that French GPs refer 6.8% of patients to NPHPs, with physiotherapists, podiatrists, and nurses accounting for 85.3% of referrals. We found higher referral rates are associated with older, retired patients, with multiple health problems, and longer consultation durations. Specific associations and trends were observed for referrals to nurses, physiotherapists, and podiatrists.

To our knowledge, no data are available from other countries that may be directly compared to ours in terms of referral rates. Publications concerning NPHP referrals mostly rely on declarative data, which are subject to different biases (such as memory or social desirability), rather than observational practice-based data, and do not report consultation-scale data that would allow GP referral rates to be estimated [24, 25].

In France, the health system requires a GP to prescribe physiotherapist, podiatrist, or nurse treatments which the national health insurance then reimburses. This would explain why GPs refer to these three NPHPs most often, at least explicitly, as opposed to a pharmacist, a midwife, or a dentist for whom formal referral is not needed. GPs in France prescribe medication to patients in 80.7% of consultations, making pharmacists possibly the most referred to profession [26], but this referral is not explicitly discussed with the patient. Importantly, the GP referral process does not cover visits to nutritionists, psychologists, or osteopaths, which the French national health insurance will not reimburse. Patients most often decide to consult these non-reimbursed professionals either upon GP advice or through a self-referral process explaining the lower formal referral rates to these NPHPs in our study.

In addition to the issue of reimbursement, there may be other barriers to patients consulting these non-reimbursed NPHPs. These barriers include NPHPs, such as psychologists and dieticians, often being less accessible in comparison to other health professionals since they have shorter opening hours, rarely perform home visits and there are fewer of them meaning distribution is reduced [27]. Furthermore, acceptability may be an issue for some patients since there is still stigma surrounding eating or weight disorders and mental health problems and patients can find these disorders difficult to accept [28–31].

GPs and nurses often support patients presenting with mental health or eating disorders, since they commonly have expertise in these areas [32–35]. Some of these patients would also benefit from psychologist or dietician consultations [36, 37]. In France, patients with chronic diseases such as diabetes can also be supported by health professional teams including GPs and public health nurses, with interesting results for some intermediate outcomes (such as glycaemia and adherence to follow-up tests). However, morbidity and mortality results are still lacking [38, 39]. Regardless of the team configuration, role clarification is essential to prevent conflicts between team members and implement effective interprofessional care [40, 41]. However, the implications of overlapping tasks for patients and health professionals remain largely unknown.

Importantly, not all patients need referring to an NPHP. GPs provide comprehensive and patient-centred care for many patients, alone or with other health professionals. They play an essential role aiming to ensure the limited available health care resources are allocated equitably to those who require further care. In this respect, we observed that increased age and multiple health problems managed were associated with high referral probability. This association has already been observed with GP referrals to dieticians and nutritionists in Australia [25] and is consistent with chronic condition and multimorbidity prevalence increasing with age [42, 43] and the increased need for multiprofessional care for these patients [44, 45]. In addition, we found consultation duration was longer (3 min on average) if a referral ensued. This may suggest that complex, comorbid patients require more referrals, or the extra duration may reflect the time needed to discuss the referral with the patient and write the referral letter.

Furthermore, the higher rate of physiotherapist referral in female patients has already been described in the literature [46]. The literature also reveals that female physicians have longer consultation durations, and are more likely to make follow-up arrangements and referrals and perform female prevention procedures [47]. This could partly be explained by physician-patient gender concordance [47].

GPs not actively creating networks with local NPHPs [48] or GPs and NPHPs not working together in multiprofessional centres [49] have been previously suggested as potential barriers to multiprofessional practice. In our study, co-location seemed to have a limited influence, if any, on referral frequency, since only podiatrist referrals were slightly higher in multiprofessional centres versus solo or mono-professional group centres. Overall, patient characteristics and clinical situations impact referral frequency more than GP-related factors suggesting that patients who need referral most are indeed those who are most often referred.

Several authors describe the referral process as a first step in interprofessional collaboration or teamwork [50, 51]. It is therefore important to acknowledge that our study does not enable in-depth analysis of the degree of collaboration, or the conditions required to implement efficient interprofessional practice in primary care. Furthermore, GP practices may not always be aligned with optimal care and observing practice referrals is not sufficient to determine what their referral frequency should be. However, improved understanding of the frequency and factors associated with GP referral to NPHPs provides useful information for health policy development support including planning for future healthcare workforce requirements and implementing interprofessional teams in primary care.

Although the ECOGEN study is slightly dated, to our knowledge, it is the only study which has reported data on NPHP referrals in France and equivalent data do not exist for other countries. Study sample size is large with 128 GPs and 20,613 patient consultations. Included GPs were representative of French GPs in terms of age, gender, practice location and annual consultation numbers [17, 26]. However, all participating GPs were GP internship supervisors. Compared with other GPs, they have similar continuing professional development participation rates, and their patient characteristics don’t differ, but they more often work in group practice and have shorter weekly working hours. The potential impact of these differences on our results is difficult to appreciate. Furthermore, the proportion of female GPs has increased steadily since the ECOGEN study was performed and was 44.2% in 2019 [data upon request from French health insurance (CNAMTS)] versus 34% in the ECOGEN study, which may slightly influence referral frequencies.

Data collection completeness was excellent since most patients consented to participate (only 0.8% (*n* = 168) of consultations were not included due to lack of consent). Data entry was reliable as no significant difference was observed between the 4.7% double recorded consultations (mean difference: 0.002; *p* = 0.69) [17].

However, study design introduces an information bias, as only new and explicit referrals were considered in the data collection. This may underestimate referral rates, especially for nurses, where prescriptions are commonly written for several months. Furthermore, the factors associated with referral rates are based on univariate statistical analysis since this study aimed to describe GP practice from an organisational perspective. Modelling patient referral probability with a more clinical perspective would require multivariate analyses.

Beyond the general observation suggesting that national health system characteristics may be a strong determinant for NPHP referrals, our data are specific to France and applicability to other settings is not possible without caution. Should data from other countries become available, it would be interesting to compare them with the ECOGEN data and determine the similarities or differences according to health care system organisation, including NPHP reimbursement. Future investigations should also explore the implications of receiving care from different health professionals, according to specific situations and needs, as well as implications for providers who are potentially filling in for specialist care due to limited accessibility. Finally, future research should address modalities of interprofessional collaboration and teamwork rather than just referrals.

## Conclusions

In France, GPs refer around 1 out of 15 patients (6.8%) to NPHPs. Referral frequency is associated with patient characteristics and clinical situations more than GP-related factors, suggesting that patients needing referral most are most often referred. Physiotherapists, podiatrists, and nurses are the most common referrals because the French national health insurance only reimburses treatment costs for these three NPHPs upon GPs referral. Health system legislation and NPHP reimbursement appear to be strong determinants for NPHP referrals. This means there is room for change if health policies aim to support multiprofessional care development.

## Data Availability

The datasets analysed during the current study are available from the corresponding author on reasonable request.

## Abbreviations

GP: General practitioner
NPHP: Non-physician health professional
ICPC-2: International Classification of Primary Care, 2nd edition
